# The RNA-binding proteins *Zfp36l1* and *Zfp36l2* act redundantly in myogenesis

**DOI:** 10.1186/s13395-018-0183-9

**Published:** 2018-12-07

**Authors:** Hema Bye-A-Jee, Dhamayanthi Pugazhendhi, Samuel Woodhouse, Patrick Brien, Rachel Watson, Martin Turner, Jennifer Pell

**Affiliations:** 1 Laboratory of Cell Signalling, The Babraham Institute, Cambridge, UK; 2 Laboratory of Lymphocyte Signalling and Development, The Babraham Institute, Cambridge, UK; 3 Department of Pharmacology, University of Cambridge, Tennis Court Road, Cambridge, UK; 4 Present address: European Molecular Biology Laboratory, European Bioinformatics Institute (EMBL-EBI), Wellcome Genome Campus, Hinxton, Cambridge, UK; 5 Present address: Illumina Cambridge Ltd, Chesterford Research Park, Little Chesterford, Saffron Walden, Essex UK; 6 Present address: University of Oxford Medical School, Medical Sciences Division, University of Oxford, John Radcliffe Hospital, Oxford, UK; 7 Present address: Human Genetics, Wellcome Trust Sanger Institute, Wellcome Genome Campus, Hinxton, Cambridge, UK

**Keywords:** RNA, RNA-binding proteins, Satellite cells, Skeletal muscle stem cells, Regeneration, Injury, Zfp36L1, Zfp36L2

## Abstract

**Background:**

Members of the ZFP36 family of RNA-binding proteins regulate gene expression post-transcriptionally by binding to AU-rich elements in the 3’UTR of mRNA and stimulating mRNA degradation. The proteins within this family target different transcripts in different tissues. In particular, ZFP36 targets myogenic transcripts and may have a role in adult muscle stem cell quiescence. Our study examined the requirement of ZFP36L1 and ZFP36L2 in adult muscle cell fate regulation.

**Methods:**

We generated single and double conditional knockout mice in which *Zfp36l1* and/or *Zfp36l2* were deleted in Pax7-expressing cells. Immunostained muscle sections were used to analyse resting skeletal muscle, and a cardiotoxin-induced injury model was used to determine the regenerative capacity of muscle.

**Results:**

We show that ZFP36L1 and ZFP36L2 proteins are expressed in satellite cells. Mice lacking the two proteins in Pax7-expressing cells have reduced body weight and have reduced skeletal muscle mass. Furthermore, the number of satellite cells is reduced in adult skeletal muscle and the capacity of this muscle to regenerate following muscle injury is diminished.

**Conclusion:**

ZFP36L1 and ZFP36L2 act redundantly in myogenesis. These findings add further intricacy to the regulation of the cell fate of Pax7-expressing cells in skeletal muscle by RNA-binding proteins.

**Electronic supplementary material:**

The online version of this article (10.1186/s13395-018-0183-9) contains supplementary material, which is available to authorized users.

## Background

Paired box transcription factors 3 and 7 (Pax3 and Pax7, respectively) are co-expressed in the dermomyotome during mouse embryonic development [1, 2]. Their expression gives rise to myogenic progenitor cells which are necessary for myogenic development. As critical regulators, Pax3 is required for embryonic myogenesis, whilst Pax7 is predominantly required for adult myogenesis and specifies the adult stem cell population in muscle [3–5]. The identity of the cells that arise from the myogenic progenitor cells during embryonic and adult myogenesis may be determined by the expression of myogenic regulatory factors (MRFs) that are expressed downstream of Pax3 and Pax7. In adult skeletal muscle, satellite cells express Pax7 and they are the adult stem cell population [5]. They largely reside in a mitotically quiescent state between the basal lamina and plasma membrane of myofibres [6, 7]. Typically, in response to growth cues, tissue injury or disease, signals from the surrounding environment, and from within the specialised stem cell niche, promote exit from the quiescent state and induce satellite cell activation [8–11]. Following activation, satellite cells proliferate and either enter the myogenic differentiation programme or self-renew to re-establish the quiescent satellite cell pool [8–10]. Knowledge of the mechanisms that maintain adult stem cells within tissues in an undifferentiated state, and control the differentiation or self-renewal fate of these cells, is important for understanding tissue homeostasis and may have application in the emerging field of regenerative medicine.

Genetic programmes act transcriptionally and post-transcriptionally to achieve stringent regulation of the development, specificity and replenishment of skeletal muscle. At the level of transcription, MRFs bind to the promoters of myogenic determination genes to initiate gene expression and thus govern the transition from the muscle precursor cell or satellite cell to multinucleated myofibre throughout development [12, 13]. Post-transcriptional regulation by alternative splicing and polyadenylation, or by control of mRNA translation and degradation, also regulates gene expression in satellite cells. Specifically, the interaction between cis-acting elements within the mRNA and trans-acting elements, such as microRNAs and RNA-binding proteins (RBPs), regulates transcript stability and translation. There is accumulating evidence to demonstrate that RBPs are necessary for normal muscle physiology [14, 15]. In particular, RBPs including HuR, AUF1, ZFP36 and KSRP bind to AU-rich elements to modulate the expression of numerous myogenic regulatory factors that function at different phases of myogenesis to ensure normal skeletal muscle development. The overlapping roles of these RBPs indicate that the coordination between RBPs is necessary for the normal progression of myogenesis [16, 17]. Collectively, this highlights the importance of RBPs in establishing cell identity during the normal myogenic process.

*Zfp36* encodes tristetraprolin (TTP) the prototype of a small family of RBPs, called the ZFP36 family, that are characterised by highly conserved tandem CCCH zinc-finger RNA-binding domains [18]. ZFP36 is a RBP that promotes RNA decay and negatively regulates the expression of the myogenic regulatory factor MyoD by binding to the 3’UTR of MyoD mRNA [1]. Mouse satellite cells from *Zfp36*-deficient mice express increased amounts of MyoD and display impaired satellite activation, demonstrating a role for ZFP36 in the maintenance of quiescence [1]. The functions of the ZFP36L1 and ZFP36L2 family members have not been evaluated in skeletal muscle stem cell fate, but have been shown to act redundantly to promote quiescence during lymphocyte development [19–21]. ZFP36L1 has been implicated in the persistence of the marginal zone B lymphocyte population [22] and ZFP36L2 in the maintenance of the haematopoietic stem cell pool [23]. To date, the roles of ZFP36L1 and ZFP36L2 in specific myogenic cell populations have not been studied. Here, we demonstrate that loss of both ZFP36L1 and ZFP36L2 in Pax7-expressing cells results in a reduced number of satellite cells in adult mice, and furthermore, provide evidence that these genes promote regeneration following muscle injury. Mechanistically, we suggest that ZFP36L1 and ZFP36L2 may act redundantly in determining the cell fate of Pax7-expressing cells during the normal myogenic process, and we provide additional insight into the complexity of the regulation of skeletal muscle myogenesis by RBPs.

## Methods

### Mice

Mice were bred and maintained in the Babraham Institute Biological Support Unit under Specific Opportunistic Pathogen Free (SOPF) conditions. After weaning, mice were transferred to individually ventilated cages with 1–5 mice per cage. Mice were fed CRM (P) VP diet (Special Diet Services) *ad libitum* and received environmental enrichment. Mario Capecchi (University of Utah) provided transgenic mice expressing Cre-recombinase under the control of the Pax7 promoter (Pax7Cre) [24]. The *Zfp36l1*^tm1.1Tnr^ and *Zfp36l2*^tm1.1Tnr^ targeted conditional alleles were generated at the Babraham Institute [19]. Interbreeding generated mice on the C57BL/6 genetic background in which either or both floxed alleles were present with Pax7Cre. For simplicity, these will be referred to as Zfp36L1-P, Zfp36L2-P and Zfp36L1/L2-P for Pax7Cre*Zfp36l1*^*fl/fl*^, Pax7Cre*Zfp36l2*^*fl/fl*^ and Pax7Cre-*Zfp36l1*^*fl/fl*^*Zfp36l2*^*fl/fl*^, respectively. All experiments were carried out without blinding or randomisation on 3–12**-**week-old mice.

### Satellite cell isolation

Satellite cells were isolated from hind limb skeletal muscles from mice using a protocol adapted from Woodhouse et al. [25]. Briefly, 6–12-week-old mice were sacrificed by rising CO_2_ and/or cervical dislocation according to Schedule One of the Home Office Protocol for the sacrifice of mice. Hind limb skeletal muscles (including gastrocnemius, plantaris, soleus, tibialis anterior (TA), extensor digitorum longus and quadriceps) were dissected from the mice. To isolate individual cells, the muscles were mechanically homogenised and further digested in a DMEM solution containing 0.12% (*w*/*v*) type II collagenase (Lorne Laboratories) and 2 mg/ml dispase II (Roche) at 37 °C with intermittent trituration using a 19G needle during the incubation. To remove excess debris, PBS was added and the slurry was passed through a 40-μM filter. The filtrate was centrifuged at 1500×*g*, and the supernatant was discarded. Red blood cells were lysed in a solution consisting of 140 mM NH_4_Cl, 2 mM Tris-HCl at pH 7.2. Lysis was stopped by addition of PBS. The remaining cells were extensively washed, and non-specific antibody binding sites were blocked with a PBS solution containing 2% foetal bovine serum (PAA; PBS/FBS). Cells in PBS/FBS were incubated with the following primary antibodies in the dark: anti-CD31-PE (Abcam), anti-CD45-PE (BD Biosciences), anti-Sca1-PE (BD Biosciences), anti-VCAM1-biotin (BD Biosciences) and anti-CD34-APC (eBioscience). The VCAM1-biotin antibody was visualised by staining with streptavidin-AlexaFluor488 conjugate (Life Technologies). Cells were finally stained with DAPI (2.5 μg/ml; Life Technologies) and sorted using a BD FACSAria III cell sorter (BD Bioscience). Antibodies against CD31, CD45 and Sca-1 were used to gate the lineage-negative cells. VCAM1- and CD34-positive satellite cells were gated and collected into Eppendorf tubes containing a high serum medium as described above. Satellite cells were subsequently cultured or lysed for protein extraction.

### Primary satellite cell culture

Primary satellite cells were plated at a density of 1.5 × 10^4^ per ml and cultured in a high serum medium containing 20% foetal bovine serum (PAA), 2 mM l-glutamine (Invitrogen), 2 ml antibiotic anti-mycotic solution (Promega) and 2.5 ng/ml basic fibroblast growth factor (Promega) in DMEM (Invitrogen). All cells were maintained using standard tissue culture techniques at 37 °C in 5% CO_2_ at a relative humidity of 100%.

### Muscle regeneration model

Muscle injury and regeneration was characterised in 6–12-week-old mice using cardiotoxin (CTX; Sigma-Aldrich). Mice were anaesthesised with isofluorane according to standard protocols. Ten micromolar CTX was prepared in PBS and 100 μl was injected into the TA muscle of isofluorane-anaesthetised mice. To act as an internal vehicle control, 100 μl of PBS was injected into the contralateral TA. Following the procedure, mice were housed and cared for as described above. Mice were sacrificed 1, 5, 10 and 25 days following injury by rising CO_2_ and/or cervical dislocation according to Schedule One of the Home Office Protocol for the sacrifice of mice and the TA muscles were harvested.

### Histology

Mice were sacrificed as described above and the TA was dissected from the ventral crest of the tibia and mounted vertically with the largest part of the TA on the base of a cork disc (Fischer). The proximal tendons of the TA were used to balance the TA upright against a needle to enable transverse cross-sectioning. The TA was coated in Tissue Tek (Leica) and snap frozen in liquid nitrogen-cooled iso-pentane (VWR). Transverse cross-sections (10 μm) were generated on a Leica Cryostat (Leica) and collected on pre-treated positively-charged glass microscope slides (VWR). Transverse cross-sections of the TA were stained with filtered Gill’s haematoxylin solution (Sigma-Aldrich) and eosin (VWR) to analyse tissue morphology, or Weigert’s haematoxylin (TCS Biosciences) and Van Gieson solution (Sigma-Aldrich) to analyse fibrosis, according to standard protocols. Sections were dehydrated in ethanol, immersed in xylene (VWR), mounted with a glass coverslip in Entellen (Merck) and air-dried under a laminar flow cupboard.

### Immunofluorescence of cryosections

TA cryosections on glass slides were fixed and permeabilised. Non-specific binding sites were blocked with an IgG blocking reagent (M.O.M; Vector Laboratories) and subsequently with a PBS solution containing 3% BSA, 5% goat serum and 0.2% (*w*/*v*) Triton X-100 (blocking buffer). The following primary antibodies were prepared in the blocking buffer and incubated overnight at 4 °C: anti-Pax7 (DSHB), anti-MyoD (Santa Cruz), anti-Myogenin (Santa Cruz) and anti-laminin α-2 chain (Enzo Life Sciences). Sections were incubated in the dark at room temperature with the fluorescence-conjugated secondary antibodies AlexaFluor 488 (donkey anti-rabbit; Life Technologies), AlexaFluor 568 (goat anti-mouse; Life Technologies) and/or AlexaFluor 633 (goat anti-rat; Life Technologies) in blocking buffer. Sections were washed in PBS/TX. To stain the nuclei, DAPI was diluted in PBS/TX and incubated with the sections in the dark at room temperature. The sections were washed in PBS/TX and then mounted with glass coverslips using Vectashield, a hard set mounting medium (Vector Laboratories). Slides were dried in the dark at room temperature.

### Western blotting

Proteins were extracted and Western blotting was performed as described in [26]. The following primary antibody dilutions were prepared in a PBS solution (0.1 M phosphate buffer was used throughout) containing 0.2% I-Block (Applied Biosystems) and 0.05% Tween-20: anti-BRF1/2 (Cell Signalling Technologies), anti-pan ZFP36-family (SB1/30.13) and anti-β-actin (Abcam). The following secondary antibodies were diluted in a PBS solution containing 0.05% Tween-20: goat anti-rabbit-HRP conjugated (Biorad) or donkey anti-rat-HRP conjugated (Abcam).

### Statistical analysis and imaging

The statistical tests used are indicated in the figure legends. A minimum of three replicates were performed for each experiment, and data are presented as means ± SEM. All images were acquired using a × 40 objective lens on an Olympus confocal microscope, and quantification was performed using ImageJ software (NIH).

## Results and discussion

**Fig. 1:**
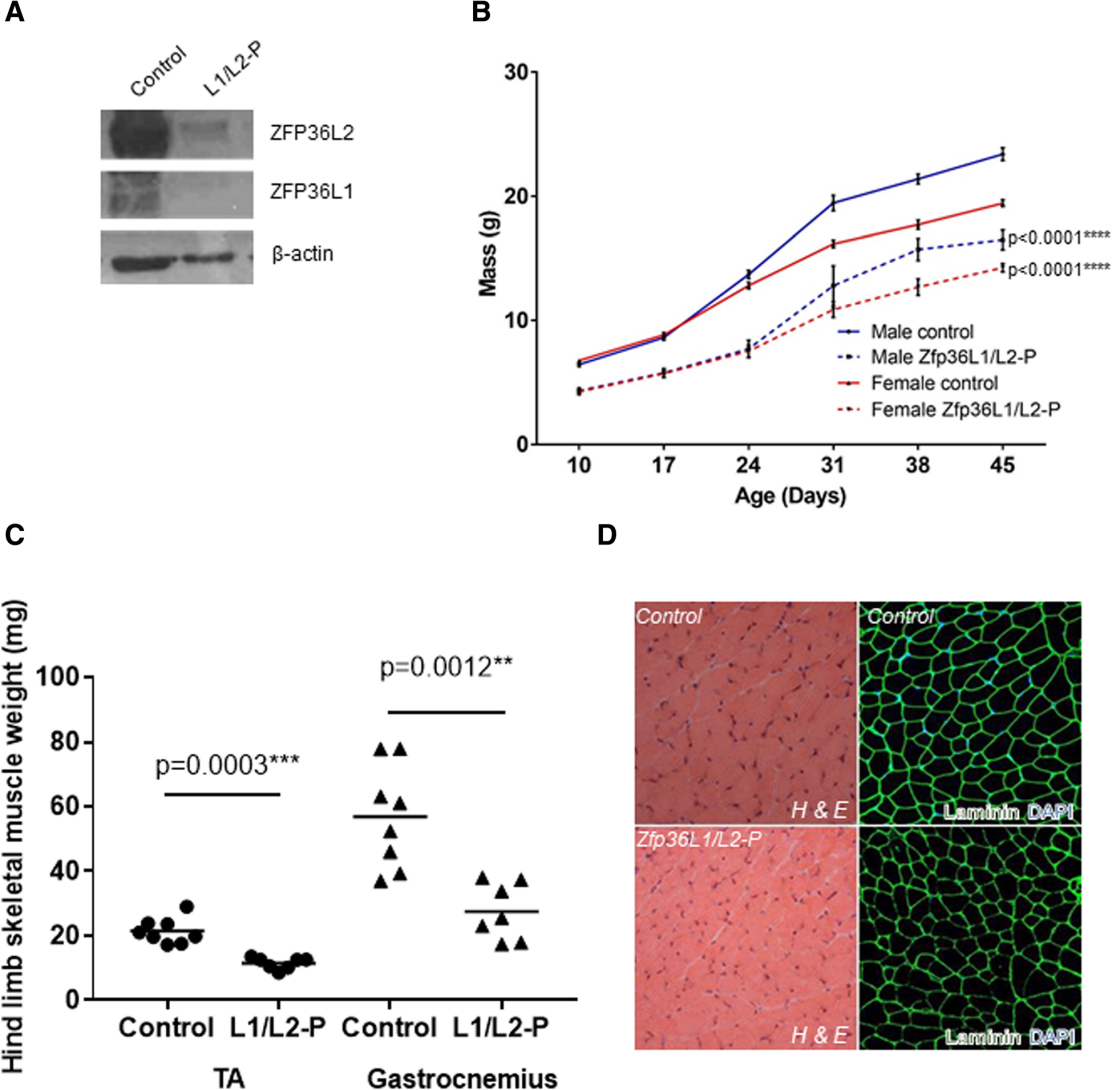
ZFP36L1 and ZFP36L2 are both required for whole body growth. Characterisation of Zfp36L1/L2-P mice. Controls represent Cre-negative littermates. **a** Western blot showing the ablation of ZFP36L1 and ZFP36L2 in isolated satellite cells from Zfp36L1/L2 mice (see also Additional file 1). **b** Weights of male and female Zfp36L1/L2-P and control mice measured from 10 days to 45 days of age. Error bars represent SEM, *p* < 0.0001**** measured by two-way ANOVA with Tukey’s multiple comparison test, *n* = 10. **c** Weights of tibialis anterior (TA) and gastrocnemius hind limb skeletal muscle from 3- to 4-week-old Zfp36L1/L2-P and control mice in milligrammes. Significance was measured by unpaired, two-tailed Mann Whitney test, *n* = 8 for control; *n* = 7 for Zfp36L1/L2-P. **d** Transverse cross sections of TA muscles were stained with haematoxylin (H; myofibre; pink) and eosin (E; nuclei; purple), and immunostained with antibodies against laminin (green), an extracellular matrix protein to delineate the periphery of the individual muscle fibres and with DAPI (blue), a nuclear marker. Scale bars: 100 μm. H and E images were acquired using a × 20 objective lens, and immunofluorescence images were acquired using a × 40 objective lens. Images representative of *n* = 3

The role of RNA-binding proteins in controlling cell fate is emerging as an important mechanism. In mice, the transcripts encoding the three ZFP36 family members are expressed in the skeletal muscle system and, more specifically, are expressed to varying degrees in quiescent and activated satellite cells [1]. By the Western blotting of proteins from isolated satellite cells from mouse adult skeletal muscle, we found that both ZFP36L1 and ZFP36L2 are expressed in this myogenic cell population (Fig. 1a; the results of a second independent experiments are shown in Additional file 1). Germline knockout mice for *Zfp36l1* and *Zfp36l2* exhibit severe developmental and growth defects and as a result *Zfp36l1* knockout mice die *in utero* between E8 and E12 and *Zfp36l2* knockout mice die within 2 weeks of birth [23, 27–29]. Therefore, we adopted a conditional tissue-specific knockout approach and used Pax7Cre to delete *Zfp36l1* and/or *Zfp36l2* in Pax7-expressing cells. In mice, skeletal muscle progenitor cells arise in the dermomyotome during E9 and E12 of embryonic development, and specifically, Pax7 is first expressed in muscle progenitor cells in the central regions of the dermomyotome at around E10 [2, 4, 30, 31]. In our model, *Zfp36l1* and/or *Zfp36l2* would therefore be deleted in Pax7-expressing progenitor cells during the development of the dermomyotome, as well as in Pax7-expressing cells in adults.

Mice with targeted mutation of either RBP in their Pax7-expressing cells were viable and did not display any whole body growth or muscle mass defects (Additional file 2). Thus, the expression and activity of Pax7Cre, or the loss of *Zfp36l1* or *Zfp36l2*, do not prevent mouse development or affect whole body growth. Mice lacking both *Zfp36l1* and *Zfp36l2* in Pax7-expressing cells (hereafter called Zfp36L1/L2-P) were viable, but were severely growth-retarded compared to Cre-negative littermates (hereafter called control; Fig. 1b). Whole body growth retardation was apparent from 3 weeks of age and continued to adulthood in both male and female mice (Fig. 1b). Furthermore, both the TA and gastrocnemius muscles from Zfp36L1/L2-P mice were significantly reduced in weight compared to the same muscles from the control mice. Satellite cells isolated from Zfp36L1/L2-P adult mice contained no detectable ZFP36L1 or ZFP36L2 protein indicating effective ablation of both proteins (Fig. 1a and Additional file 1). However, further examination of the embryonic developmental stages from when Pax7 is first expressed is required to determine when precisely the *Zfp36l1* and *Zfp36l2* genes are deleted and any effects of this on the developing embryo. We did not establish whether ZFP36 was expressed in the isolated satellite cells from Zfp36L1/L2-P mice, but at the genetic level it was unable to compensate for the loss of *Zfp36l1* and *Zfp36l2*, and support whole body and skeletal muscle growth.

Transverse cross sections of the TA from Zfp36L1/L2-P mice stained with haemotoxylin and eosin (H and E), and immunostained with anti-laminin, indicated that the myofibre organisation and architecture were similar to controls, and although this was not quantified, we observed a mixture of small and large myofibres (Fig. 1d). Furthermore, all nuclei were peripheral and located around the myofibres, and similarly to controls, there was no apparent on-going muscle degeneration or regeneration. Quantification of myofibre size and the distribution of myofibre types may provide further understanding of the roles of ZFP36L1 and ZFP36L2 on adult skeletal muscle architecture. Taken together, these data are consistent with a requirement for *Zfp36l1* and *Zfp36l2* to promote whole body growth and sustain skeletal muscle development in adults. Studies have demonstrated that Pax7-expressing cells contribute little to embryonic development, but show that Pax7 expression is required for adult myogenesis and specification of satellite cells [3, 5, 32]. Further work is required to determine whether the observed phenotypes are due to defects in the Pax7 cell population at earlier developmental stages.

**Fig. 2:**
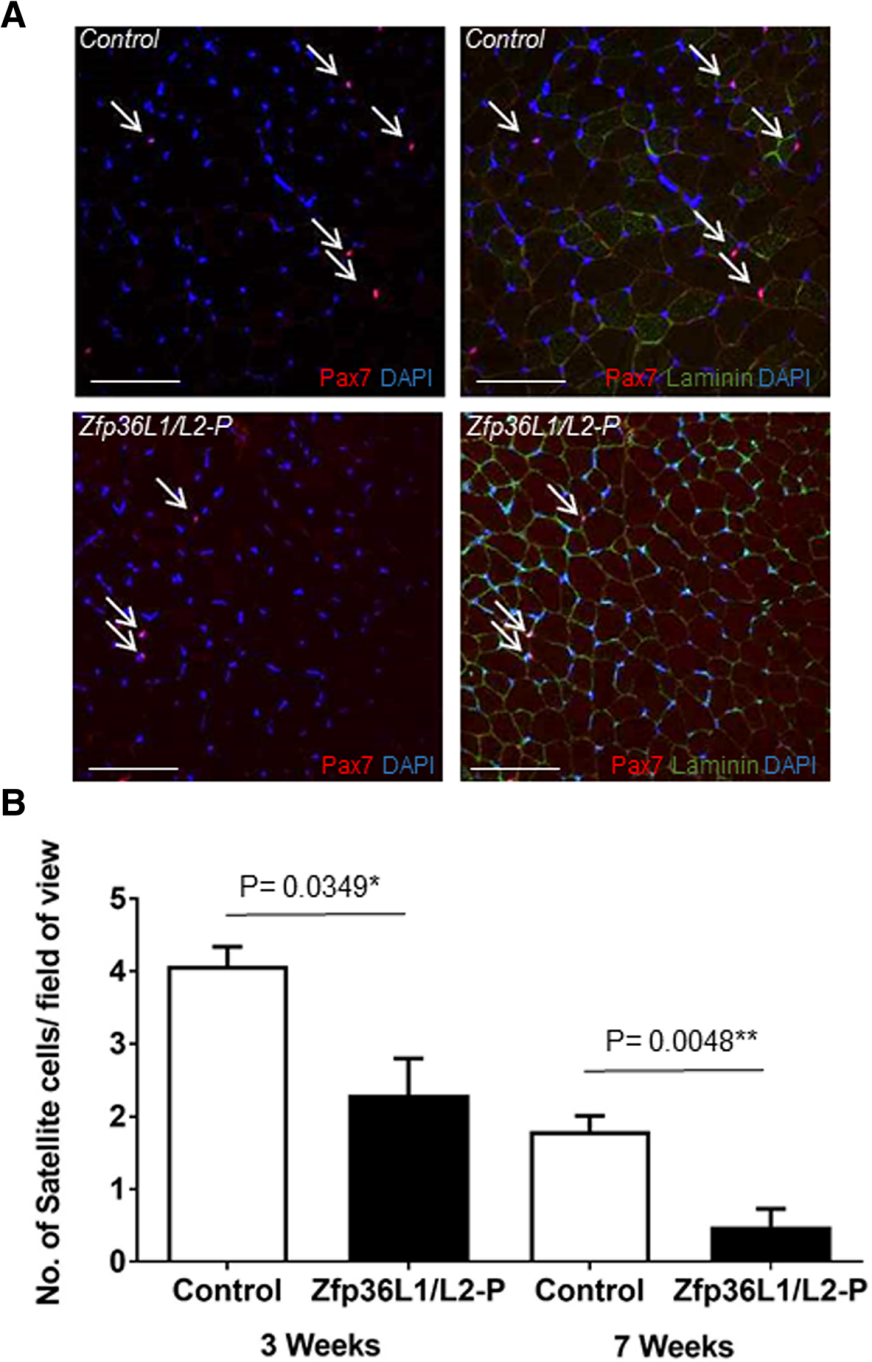
Deleting ZFP36L1 and ZFP36L2 in Pax7-expressing cells reduces the number of satellite cells in TA muscles. Transverse cross sections from 3- to 4-week-old Zfp36L1/L2-P and control mice. Controls represent Cre-negative littermates. **a** Sections were immunostained with antibodies against Pax7 (red), a marker of adult satellite cells, laminin (green), an extracellular matrix protein to delineate the periphery of the individual muscle fibres, and with DAPI (blue), a nuclear marker. Open arrows indicate Pax7+ cells. Scale bars: 100 μm. **b** Average number of satellite cells in TAs from control and Zfp36L1/L2-P mice. Error bars represent SEM, significance measured by unpaired two-tailed Student’s *t* test, *n* = 3. Calculations from 10 fields of view per experiment

In adults, studies suggest that a decrease in myofibre volume may be related to a decline in nuclei number and more specifically a decrease in satellite cells, which could delay myonuclei turnover [33]. As satellite cells are required for post-natal growth, we explored this possibility by assessing whether *Zfp36l1* and *Zfp36l2* could regulate the satellite cell population. Transverse cross sections of the TA muscle were immunostained with anti-Pax7 and were used to quantify the number of satellite cells per field of view (Fig. 2 and Additional file 2). Quantifying and investigating satellite cell fate decisions on single myofibres is a well-established and useful method of analysing a range of satellite cell dynamics [34–36]. However, it was not possible to determine the number of satellite cells or to examine satellite cell fate in single myofibres isolated from Zfp36L1/L2-P mice. In our experiments, we found that Zfp36L1/L2-P myofibres could not be successfully cultured and satellite cells seemed to ‘wash away’. This could be indicative of an intrinsic inability of satellite cells from Zfp36L1/L2-P mice to associate with the myofibre under these conditions. Further work is needed to investigate this more thoroughly.

The deletion of *Zfp36l1* alone in Pax7-expressing cells did not result in a change in satellite cell number in TA cross sections, and deletion of *Zfp36l2* had little, if any, effect on the number of satellite cells (Additional file 2). Due to the whole body growth difference in the Zfp36L1/L2-P mice, the number of satellite cells was quantified at two post-natal developmental time points to determine if there was a change in the number of satellite cells with age. At both 3 and 7 weeks of age, the number of satellite cells per field of view was significantly decreased in TAs from the Zfp36L1/L2-P mice compared to controls (Fig. 2). Whilst we only focused on the adult population of Pax7-expressing cells, it is feasible that reduced muscle mass and reduced satellite cell number in adult skeletal muscle may be due to defects and depletion of the Pax7-expressing cell pool early in embryonic development. Nevertheless, we conclude that ZFP36L1 and ZFP36L2 are not absolutely required to establish skeletal muscle and suggest that these RBPs may have a role in Pax7-expressing cells in adult skeletal muscle. Further work is required to establish the myogenic potential of the mutant Pax7-expressing cells at all stages of development. The requirement for *Zfp36l1* and *Zfp36l2* in satellite cell function is unresolved. The data suggest there is a redundant requirement for *Zfp36l1* and *Zfp36l2* for the expansion of progenitor cells and/or for the progression through the myogenic programme. Redundancy between *Zfp36l1* and *Zfp36l2* has been demonstrated in the development of lymphocytes [19–21]. ZFP36L1 and ZFP36L2 both contain two highly conserved zinc finger domains with similar RNA-binding properties [18]. Their homology and presumably similar mRNA-binding specificities provide the simplest explanation for the observed redundancy, but this requires formal demonstration.

**Fig. 3:**
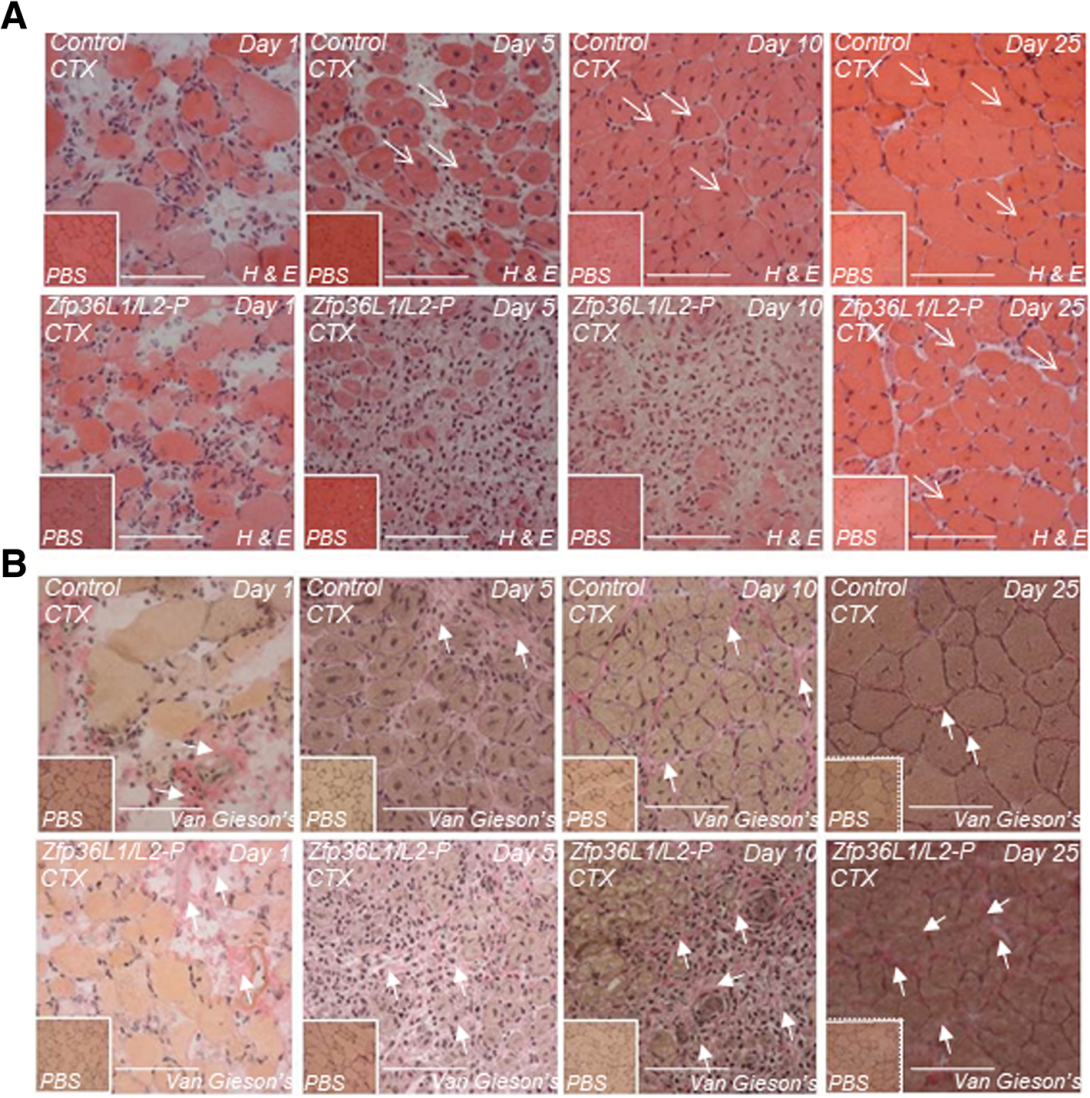
Deleting ZFP36L1 and ZFP36L2 in Pax7-expressing cells impairs skeletal muscle regeneration. The TA muscles of Zfp36L1/L2-P mice were injected with either CTX or PBS. Muscles were harvested at 1, 5, 10 or 25 days following treatment and transverse cross sections were generated. Controls represent Cre-negative littermates. **a** Sections were stained with haematoxylin (H; myofibre; pink) and eosin (E; nuclei; purple) to assess the skeletal muscle architecture. Open arrows identify centrally located nuclei, an indication of muscle regeneration. **b** Sections were Van Gieson’s stain to determine the extent of fibrosis. Closed arrows indicate fibrotic tissue (red). Insets of respective PBS-injected, uninjured contralateral TA muscles are in the left-hand corner of each image. Scale bars: 100 μm. Images representative of *n* = 3 at each time point

In addition to a requirement for post-natal growth, satellite cells also have a role in muscle regeneration following injury [25, 37–40]. To examine the regenerative capacity of skeletal muscle from Zfp36L1/L2-P mice, 6–12-week-old mice were challenged with cardiotoxin (CTX), an agent that induces muscle degeneration, but leaves satellite cells intact (Fig. 3 and Additional file 3). CTX was administered locally into the TA muscle, and a PBS “vehicle control” was administered into the contralateral muscle. Muscles were recovered at 1, 5, 10, or 25 days following treatment, and transverse cross sections were stained with H and E to assess the extent of damage to the skeletal muscle architecture (Fig. 3a and Additional file 3), and Van Gieson’s stain, which stains collagen and is an indicator of fibrosis (Fig. 3b). TA muscles from both mutant and control mice that had been injected with the vehicle control did not exhibit any injury and there was no visible change in myofibre arrangement or obvious fibrosis throughout the injury time course (Fig. 3 and Additional file 3). TAs injected with the vehicle control was thus valid uninjured controls. H and E staining of CTX-injured TAs from mice lacking either *Zfp36l1* or *Zfp36l2* in Pax7-expressing cells was indistinguishable from controls (Additional file 3).

TAs from control and Zfp36L1/L2-P mice injected with CTX demonstrated striking morphological changes characterised by myofibre degeneration and an increased number of nuclei, indicative of infiltrating leukocytes and fibrosis (Fig. 3). Typically, in the injured control TAs at 1 day post-injury, myofibres dissociated from their regular arrangement; at 5 and 10 days post-injury, there was further myofibre disruption and increased leukocyte infiltration (inferred from the staining of nuclei) as the muscle degenerated; and at 25 days post-injury, myofibre regeneration was apparent by the presence of myofibres that harboured nuclei, which were centrally located, and fewer nuclei of infiltrating cells (Fig. 3). TAs from Zfp36L1/L2-P mice at day 1 following muscle injury, appeared similar to injured TAs from control mice. At 5 and 10 days post-injury, muscle regeneration appeared delayed and there was increased collagen staining in between myofibres compared to controls (Fig. 3b). At 25 days post-injury, there were centrally located nuclei within myofibres, indicating regeneration. However, this regeneration appeared to be reduced in comparison to control TAs (Fig. 3a). As we deleted ZFP36L1 and ZFP36L2 in Pax7-expressing cells, it is possible that the impaired regeneration may be due to defects in the function of myogenic cells derived from Pax7-expressing progenitor cells.

**Fig. 4:**
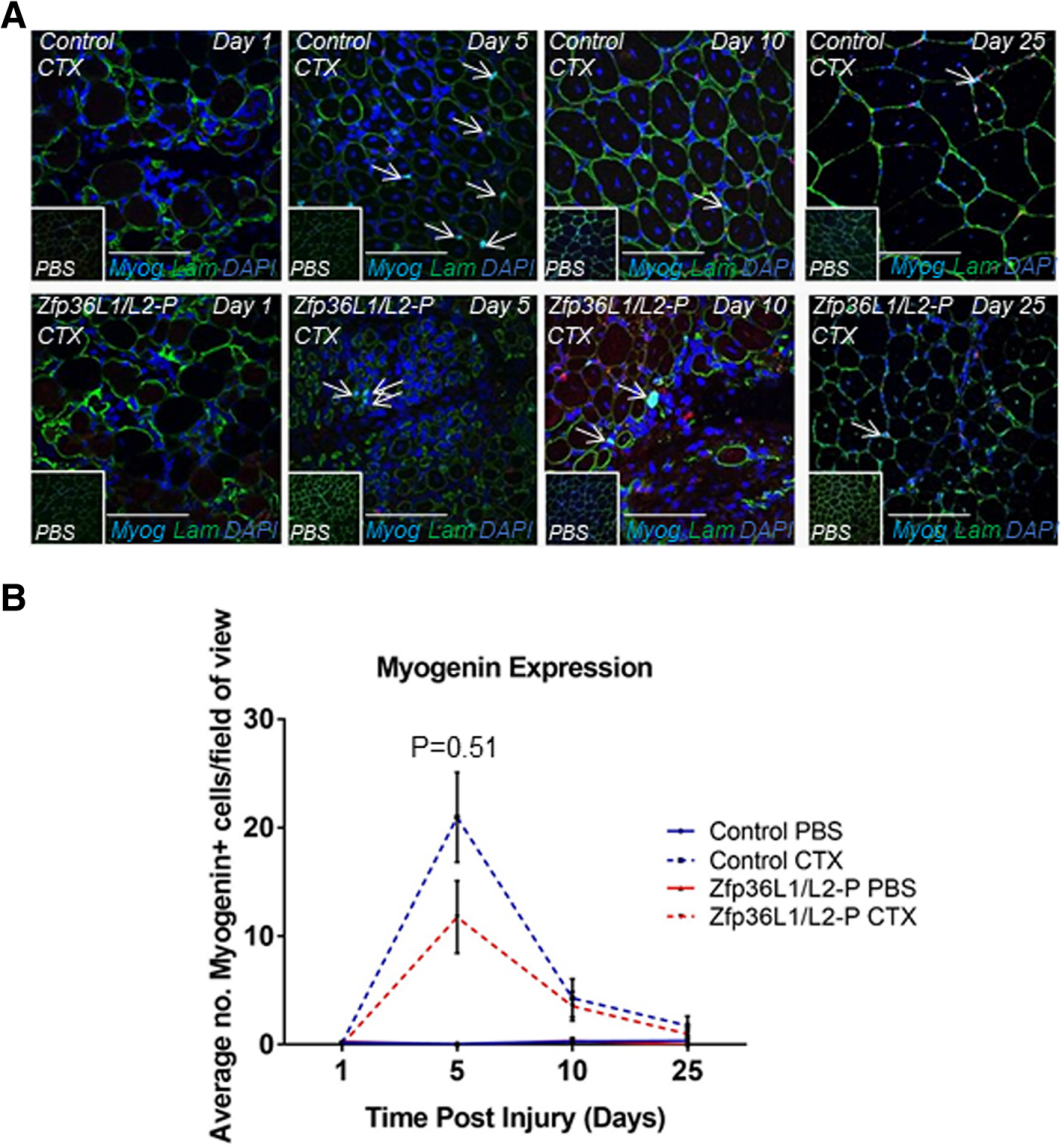
Terminal differentiation can occur independently of ZFP36L1 and ZFP36L2. Transverse cross sections of injured TAs from Zfp36L1/L2-P mice were immunostained with antibodies for Pax7 (red), myogenin (myog; turquoise), laminin (lam; green), and counterstained with the nuclear marker DAPI (blue). Controls represent Cre-negative littermates. **a** Representative images of injured control and Zfp36L1/L2-P TAs. Insets represent PBS-injected, uninjured contralateral TA muscles. Open arrows indicate myogenin+ myogenic cells (myog; turquoise). Scale bars: 100 μm. **b** Average number of myogenin+ cells present in injured control and Zfp36L1/L2-P TAs during the injury time course. Images were acquired using a × 40 objective lens. Calculations were based on 10 fields of view per experiment. Error bars represent the SEM, two-way ANOVA adjusted for multiple comparisons with Sidak’s post hoc test, *n* = 3

To investigate the requirement for *Zfp36l1* and *Zfp36l2* further, transverse cross sections from injured control and Zfp36L1/L2-P were immunostained with antibodies against myogenin (Fig. 4a), which is required for myoblasts to differentiate further into myofibres. In injured TAs from control and Zfp36L1/L2-P mice, there was a peak in the number of myogenin+ cells at the 5 day time point (Fig. 4b). At the 10 and 25 day time points, the numbers of myogenin+ cells in the injured TAs from Zfp36L1/L2-P mice were similar to those in the injured control TAs (Fig. 4b). Whilst we are unable to exclude the possibility that a difference might have been found with a larger sample size, there was little effect, if any, of the deletion of *Zfp36l1* and *Zfp36l2* on terminal differentiation at day 5. We show that muscle from Zfp36L1/L2-P mice has a diminished regenerative capacity and suggest that this may reflect the reduced availability or function of satellite cells.

**Fig. 5:**
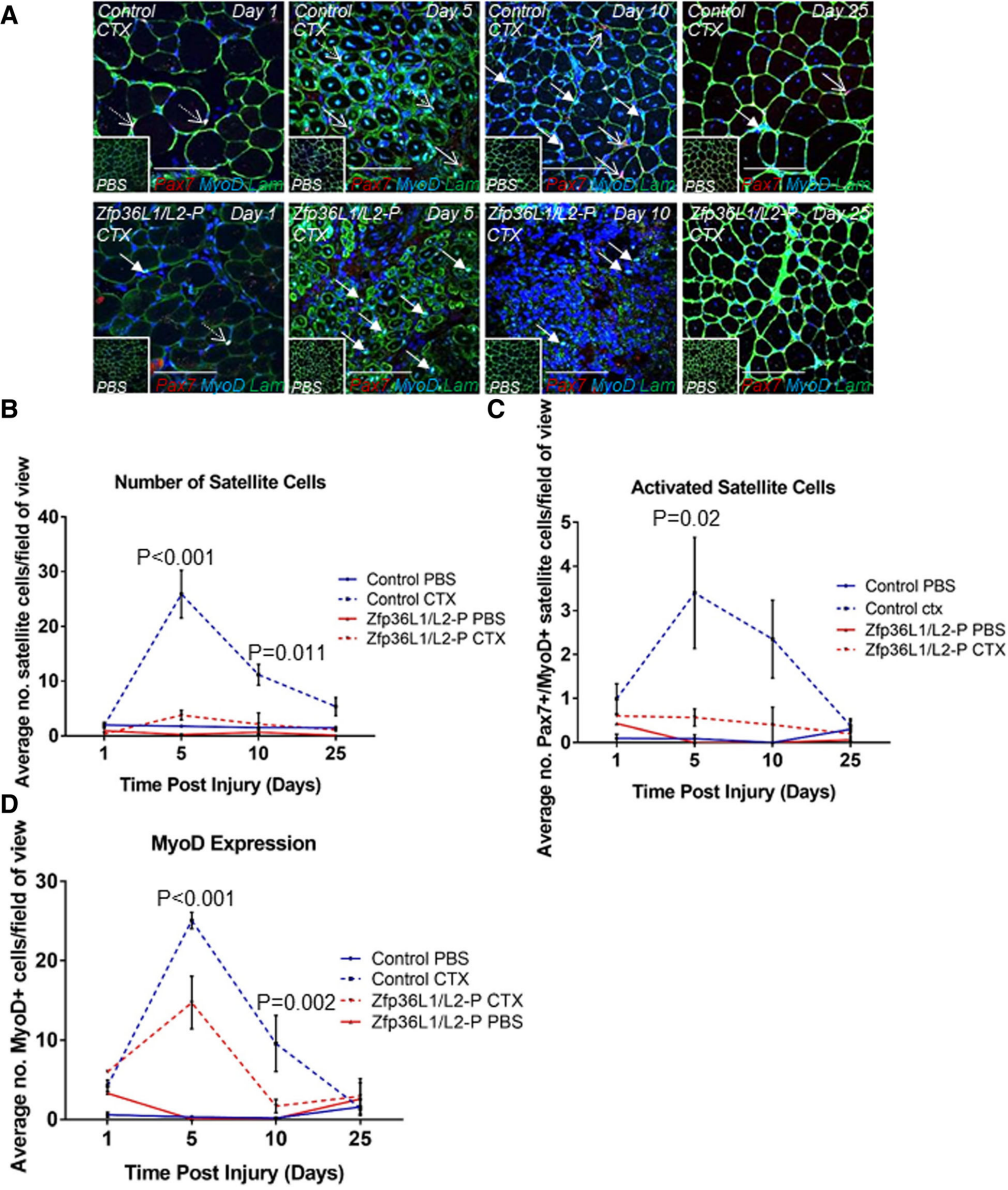
Deleting ZFP36L1 and ZFP36L2 in Pax7-expressing cells impairs expansion of the satellite cell pool during skeletal muscle regeneration. Transverse cross sections of injured TAs from Zfp36L1/L2-P mice were immunostained with antibodies for Pax7 (red), MyoD (turquoise), Laminin (lam; green), and counterstained with the nuclear marker DAPI (blue). Activated cells were co-stained with Pax7 and MyoD (white). Controls represent Cre-negative littermates. **a** Representative images of injured control and Zfp36L1/L2-P TAs. Insets represent PBS-injected, uninjured contralateral TA muscles. Open arrows indicate Pax7+ satellite cells (red); closed arrows indicate MyoD+ myogenic cells (turquoise), and dotted arrows indicate Pax7+MyoD+ activated satellite cells (white). Scale bars: 100 μm. **b** Average number of satellite cells present in injured control and Zfp36L1/L2-P TAs during the injury time course. **c** Average number of activated cells present in injured control and Zfp36L1/L2-P TAs during the injury time course. **d** Average number of MyoD+ cells present in injured control and Zfp36L1/L2-P TAs during the injury time course. Images were acquired using a × 40 objective lens. Calculations from 10 fields of view per experiment. Error bars represent the SEM, two-way ANOVA adjusted for multiple comparisons with Sidak’s post hoc test, *n* = 3

To assess the satellite cell population, CTX-injured TAs were stained during the injury time course with anti-Pax7 to identify quiescent satellite cells and self-renewed satellite cells, and anti-MyoD to identify muscle lineage committed cells (myoblasts). Cells co-staining with anti-Pax7 and anti-MyoD were considered to be activated (Pax7+MyoD+) satellite cells (Fig. 5a). At each time point, we quantified the number of Pax7+, MyoD+ and Pax7+MyoD+ cells in sections from injured and uninjured TAs in control and Zfp36L1/L2-P mice. In the TAs from control mice at days 5 and 10 post-injury, the Pax7+ satellite cell pool expanded (Fig. 5b), the number of activated (Pax7+MyoD+) satellite cells increased (Fig. 5c), and there was an increased number of MyoD+ cells (Fig. 5d). At 25 days post-injury, the number of Pax7+ satellite cells returned to uninjured levels and the number of activated (Pax7+MyoD+) satellite cells and MyoD+ cells reduced to nearly that of uninjured TAs. This suggests the return of satellite cells to quiescence and almost complete regeneration (Fig. 5b). By contrast, in injured TAs from Zfp36L1/L2-P mice, the pool of Pax7+ satellite cells did not expand, and there were negligible numbers of activated (Pax7+MyoD+) satellite cells (Fig. 5b, c). There was an increase in the number of MyoD+ cells at the 5 day post-injury time point (Fig. 5d). This increase in MyoD+ cells may contribute to the capacity of injured TAs from Zfp36L1/L2-P mice to undergo regeneration.

In our model, *Zfp36l1* and *Zfp36l2* are deleted in Pax7-expressing cells throughout development; thus, we can not rule out the possibility that the regenerative defect could be as a result of a requirement for the RBPs in Pax7-expressing cells in the dermomyotome and/or Pax7-derived myogenic cells. Here, we used the Pax7Cre mouse line to address roles for ZFP36L1 and ZFP36L2 in myogenesis. In future, the utilisation of genetic tools which permit elective deletion of *Zfp36l1* and *Zfp36l2* in Pax7-expressing cells [41, 42] would offer an opportunity to clearly define the functional properties of the adult satellite cell population independently of developmental effects. Furthermore, the measurement of mRNA abundance could reveal the effect these proteins have on gene expression and allow for mechanisms of action to be ascertained. We did not establish mRNA targets for ZFP36L1 and ZFP36L2 in our system. Interestingly, the closely related ZFP36 RBP has been shown to regulate the expression of MyoD mRNA [1], and it is feasible that ZFP36L1 and ZFP36L2 also regulate MyoD expression in myogenic cells. The application of methods that measure RNA-protein interactions in intact cells [43] could be optimised and applied to satellite cells and their myogenic derivatives from adults to identify the direct targets and thereby develop the understanding of gene regulation by RBPs in skeletal muscle.

## Conclusions

We show that *Zfp36l1* and *Zfp36l2* act redundantly to determine the number of functional Pax7-expressing cells in adult mice. The lack of a phenotypic effect of the targeting by Pax7cre of *Zfp36l1* or *Zfp36l2* alone on skeletal muscle argues against the phenotypes being due to the non-specific effects of cre. The requirement for *Zfp36l1* or *Zfp36l2* in Pax7-expressing cells is important for the growth and regenerative capacity of adult skeletal muscle. The mechanisms by which these RBPs regulate the function and fate of Pax7-expressing cells remains to be established, but in other systems, these RBP regulate quiescence [20] and cellular identity [22, 44]. Our findings should prompt further experiments designed to understand how ZFP36L1 and ZFP36L2 control cell fate decisions and the kinetics of cell turnover.

## Additional files

Additional file 1: Confirmation of ablation of ZFP36L1 and ZFP36L2 in satellite cells of Zfp36L1/L2-P mice. Western blot determining the expression of ZFP36L1 and ZFP36L2 in isolated satellite cells from Zfp36L1/L2-P and control mice. (TIF 76 kb)

Additional file 2: Characterisation of mice lacking either *Zfp36l1* or *Zfp36l2* (referred to as Zfp36L1-P or Zfp36L2-P mice). A. Weights of male and female mice measured from 10 to 45 days. Error bars represent SEM, two-way ANOVA with Tukey’s multiple comparison test, *n* = 10. B. TA and gastrocnemius muscle weights from 7-week-old male and female mice. Significance was measured by unpaired two-tailed Mann Whitney test; *n* = 6 for Zfp36L1-P and its respective control, and *n* = 8 for Zfp36L2-P and its control. Red data points indicate female mice and blue data points indicate male mice for the Zfp36L2-P graph. C. Average number of satellite cells in cross-sections of TAs from 7-week-old control, Zfp36L1-P and Zfp36L2-P mice. Error bars represent SEM, significance was measured by unpaired two-tailed Student’s *t* test; *n* = 5 for Zfp36L1-P and its respective control, and *n* = 4 for Zfp36L2-P and its control. Controls represent Cre-negative littermates. Calculations based on 10 fields of view per experiment. (TIF 201 kb)

Additional file 3: Transverse cross sections of the TA muscles from mice lacking either *Zfp36l1* or *Zfp36l2* (referred to as Zfp36L1-P or Zfp36L2-P mice), and their respective controls, recovered at 1, 5, 10 and 25 days following injection with either CTX or PBS, stained with haematoxylin (H; myofibre; pink) and eosin (E; nuclei; purple) to assess the skeletal muscle architecture. Open arrows identify centrally located nuclei, an indication of muscle regeneration. Controls represent Cre-negative littermates. Scale bars: 100 μm. Representative of *n* = 3. (TIF 843 kb)

## Abbreviations

CTX: Cardiotoxin
Lam: Laminin
Myog: Myogenin
RBP: RNA-binding proteins
TA: Tibialis anterior
TTP: Tristetraproline
