# Supplementary figures and images for: The RNA-binding proteins Zfp36l1 and Zfp36l2 act redundantly in myogenesis
Source: Skelet Muscle. 2018 Dec 7;8:37. doi: 10.1186/s13395-018-0183-9 (PMC6286576; doi:10.1186/s13395-018-0183-9)

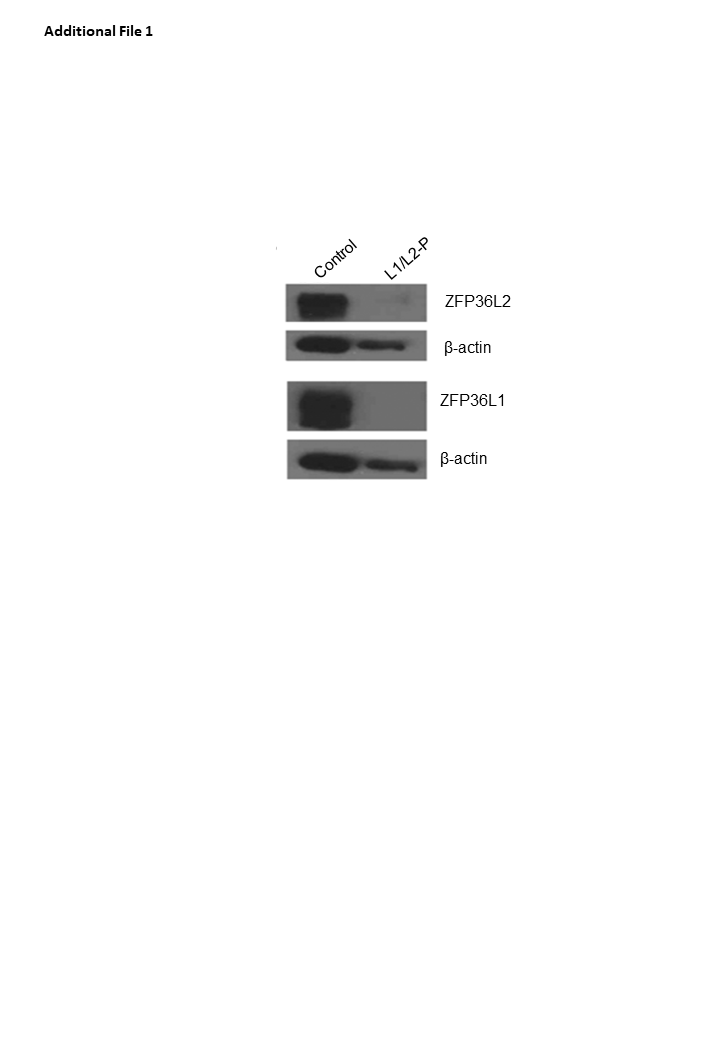

Supplement: Supplementary file 1 — Confirmation of ablation of ZFP36L1 and ZFP36L2 in satellite cells of Zfp36L1/L2-P mice. Western blot determining the expression of ZFP36L1 and ZFP36L2 in isolated satellite cells from Zfp36L1/L2-P and control mice. (TIF 76 kb) [file 13395_2018_183_MOESM1_ESM.tif]

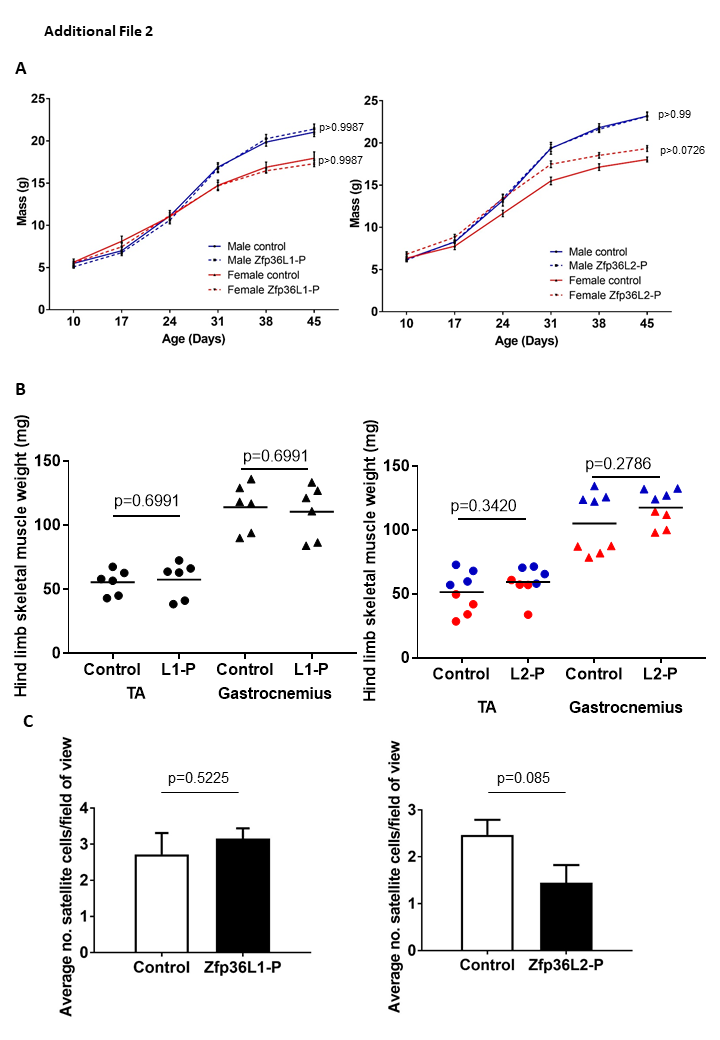

Supplement: Supplementary file 2 — Characterisation of mice lacking either Zfp36l1 or Zfp36l2 (referred to as Zfp36L1-P or Zfp36L2-P mice). A. Weights of male and female mice measured from 10 to 45 days. Error bars represent SEM, two-way ANOVA with Tukey’s multiple comparison test, n = 10. B. TA and gastrocnemius muscle weights from 7-week-old male and female mice. Significance was measured by unpaired two-tailed Mann Whitney test; n = 6 for Zfp36L1-P and its respective control, and n = 8 for Zfp36L2-P and its control. Red data points indicate female mice and blue data points indicate male mice for the Zfp36L2-P graph. C. Average number of satellite cells in cross-sections of TAs from 7-week-old control, Zfp36L1-P and Zfp36L2-P mice. Error bars represent SEM, significance was measured by unpaired two-tailed Student’s t test; n = 5 for Zfp36L1-P and its respective control, and n = 4 for Zfp36L2-P and its control. Controls represent Cre-negative littermates. Calculations based on 10 fields of view per experiment. (TIF 201 kb) [file 13395_2018_183_MOESM2_ESM.tif]

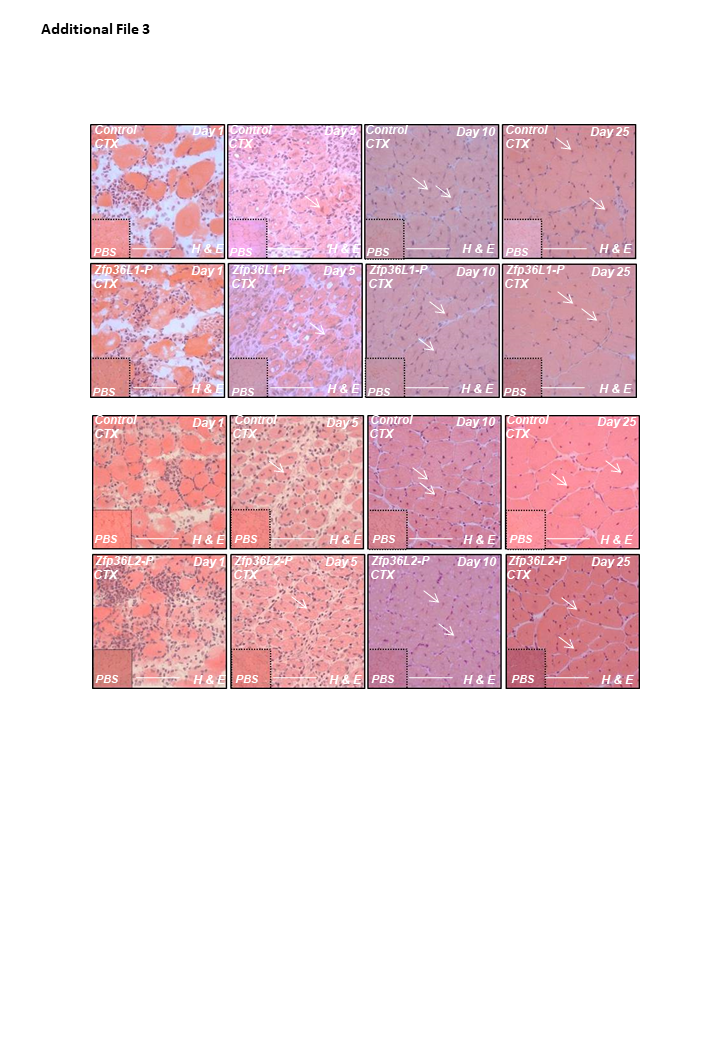

Supplement: Supplementary file 3 — Transverse cross sections of the TA muscles from mice lacking either Zfp36l1 or Zfp36l2 (referred to as Zfp36L1-P or Zfp36L2-P mice), and their respective controls, recovered at 1, 5, 10 and 25 days following injection with either CTX or PBS, stained with haematoxylin (H; myofibre; pink) and eosin (E; nuclei; purple) to assess the skeletal muscle architecture. Open arrows identify centrally located nuclei, an indication of muscle regeneration. Controls represent Cre-negative littermates. Scale bars: 100 μm. Representative of n = 3. (TIF 843 kb) [file 13395_2018_183_MOESM3_ESM.tif]
